# Low to moderate doses of 3-methylmethcathinone (3-MMC) produce analgesic effects in healthy volunteers: a proof of principle study with a designer drug

**DOI:** 10.1007/s00213-025-06798-8

**Published:** 2025-05-01

**Authors:** Johannes G Ramaekers, Johannes T Reckweg, Natasha L Mason, Kim PC Kuypers, Stefan W Toennes, Eef L Theunissen

**Affiliations:** 1 Faculty of Psychology and Neuroscience, Maastricht University, Maastricht, The Netherlands; 2 Institute of Legal Medicine, Goethe University, Frankfurt, Germany

## Abstract

3-Methylmethcathinone (3-MMC) is a synthetic cathinone that has been scheduled in many jurisdictions after it appeared on the consumer market as a designer drug or “legal high”. At present, there are no medical applications for synthetic cathinones, but in the past cathinone and other compounds that are structurally related to amphetamine have been evaluated and recognized for their intrinsic analgesic quality. The present study aimed to assess the analgesic effects of low to moderate doses (25, 50 and 100 mg) of 3-MMC in healthy volunteers (*N* = 14) in a cross-over, placebo-controlled study. Participants were repeatedly exposed to experimental pain for up to 5 h after dosing in pressure pain threshold (PPT) and cold pressor test (CPT) paradigms. A profile of mood states questionnaire was used to assess the subjective effects of 3-MMC. Overall, 3-MMC produced dose-related elevations in pressure pain threshold and reduced subjective painfulness and unpleasantness in both experimental pain models. The analgesic effects of 3-MMC were most prominent after the 50 and 100 mg dose and persisted consistently for up to 5 h after dosing. 3-MMC also produced dose-related increments in mood that were prominent at 1 h, but not at 5 h after dosing. It is concluded that 3-MMC produces prolonged analgesic effects at doses that appear low enough to avoid a challenging subjective experience and that have been associated with a benign side effect profile. The present data warrant further research into the analgesic effects of low to moderate doses of 3-MMC in patient populations.

## Introduction

3-Methylmethcathinone (3-MMC) is a synthetic cathinone that entered the recreational market of designer drugs or “legal highs” at the end of the 2000s (Ferreira et al. 2019). Designer drugs are chemically altered versions of classical drugs of abuse, created to mimic their effects while avoiding classification as illegal or controlled substances. 3-MMC is a structural analogue of the natural psychoactive substance cathinone, a psychostimulant present in the leaves of the khat shrub (Kelly 2011). Functionally, 3-MMC stimulates the release and inhibits the reuptake of monoamines, specifically dopamine, noradrenaline and serotonin, and in addition displays strong binding affinities for adrenergic receptors (Luethi et al. 2018), which are known to modulate stimulant-induced behavior (Schmidt and Weinshenker 2014). 3-MMC also binds to serotonin (5-HT)1 A and 5HT2A receptors (Luethi et al. 2018), but their functional contributions to the effects of 3-MMC are unknown. Cathinones are structurally related to amphetamines and have been reported to produce psychostimulant, psychotomimetic and cardiovascular effects as well as dependence (Soares et al. 2021). Health risk of synthetic cathinones, including 3-MMC, have been critically reviewed by public health organizations (Araujo et al. 2015; EMCDDA 2023; Sande 2016; WHO 2023), leading to controlled substance classifications in various countries (Bonson et al. 2019; Ledberg 2015).

Classification of controlled substances is generally based on their potential for abuse and dependence, medical use and safety. In the case of 3-MMC, classifications have not considered medical applications as these currently do not exist (EMCDDA 2023; WHO 2023). However, interest in cathinones as medical products did exist in the past, i.e. the 1940s and 1950s, to suppress appetite, reduce symptoms of depression or to stimulate brain activity (Kelly 2011). At present, buproprion, the N-tert-butyl analog of cathinone, is approved as an antidepressant and smoking cessation aid (Baumann et al. 2018). Likewise, amphetamines and cathinones have been long known for their intrinsic analgesic properties as shown in human (Burrill et al. 1944; Dalal and Melzack 1998) and animal studies (Bustamante et al. 2004; Dalal and Melzack 1998; Nencini et al. 1984). The first study on the analgesic effect of amphetamine in humans found a dose-related increase in the pain threshold of participants who were exposed to peak currents through a coil that was connected to a gold electrode attached to a metal filling in their teeth, and a bath of salt water in which participants kept their right hand (Burrill et al. 1944). D-amphetamine was also shown to potentiate the analgesic effects of opioids using the same experimental paradigm in healthy volunteers (Ivy et al. 1944). Later, the capacity of amphetamine-like compounds (i.e., methylphenidate) to enhance opioid-induced pain relief was also demonstrated in clinical trials in patients with post-operative pain (Forrest et al. 1977) and chronic pain (Bruera et al. 1989, 1992; Vigano et al. 1995). The analgesic effects of amphetamine and amphetamine-like compounds have been attributed to their ability to increase monoamine concentrations in the brain. In preclinical studies, the analgesic effects of cathinone were prevented by reserpine and p-chlorophenylalanine, which deplete monoamines, and by nomifensine, which prevents neuronal uptake of amphetamines (Nencini et al. 1984). Over time, the interest in the analgesic properties of amphetamine-like compounds waned, presumably because of their abuse potential and other adverse events associated with stimulant use, particularly at higher doses.

It can be expected that the analgesic properties of cathinone will also extend to compounds that are structurally related, such as 3-MMC. However, potential benefits of 3-MMC for medical applications like pain management might be outweighed by its potential to induce significant adverse events as recently stipulated by public health organizations (EMCDDA 2023; WHO 2023). Yet, such evaluations have largely focused on extreme cases of fatal and non-fatal poisonings, often involving abuse of high doses of 3-MMC in combination with other substances. While important, such cases may not be typical for health risks at low to moderate doses of 3-MMC that might suffice for producing analgesia. Safety of low to moderate doses of 3-MMC (25, 50, and 100 mg) was recently evaluated in a first in human, placebo-controlled study in healthy volunteers (Ramaekers et al. 2024). 3-MMC caused dose-dependent but mild increments in heart rate and blood pressure that were not of clinical significance. 3-MMC also produced psychostimulant effects resulting in enhanced neurocognitive task performance, and mild but transient increments in psychotomimetic effects and drug liking. Overall, stimulant effects of low to moderate doses of 3-MMC were well tolerated, suggesting that this could be a safe dose range for which potential analgesic effects might be of interest to explore.

Hence, while previous studies (Burrill et al. 1944; Bustamante et al. 2004; Dalal and Melzack 1998; Nencini et al. 1984) have focused on analgesic effects of cathinones at full doses, the current study aimed to assess the analgesic potential of 3-MMC at subthreshold doses at which (adverse) stimulant effects are negligible or mild. Analgesic properties of low to moderate doses of 3-MMC (25, 50 and 100 mg) were assessed in healthy volunteers whose objective and subjective response to experimentally induced pain as well as their subjective profile of mood were repeatedly recorded up to 5 h after dosing.

## Methods

### Participants

Fourteen participants (9 males, 5 females) entered the study, with 12 completing all treatment conditions. Two participants dropped out for reasons unrelated to treatment and were replaced. Participants’ ages ranged from 19 to 35 years (mean (SD): 22.9 (4.3)). All participants had prior experience with stimulant drugs, including methylenedioxymethamphetamine (MDMA, 11 participants), amphetamine (5 participants), cocaine (7 participants), and synthetic cathinones (4 participants). Additionally, 12 participants reported cannabis use, 9 had used psychedelics, and 4 had used ketamine. All participants consumed alcohol. Recruitment was conducted through word-of-mouth and advertisements placed in Maastricht University buildings. Each participant provided written informed consent and underwent a comprehensive medical examination, which included a physical exam, routine laboratory tests (e.g., clinical chemistry, hematology, serology and urinalysis), vital signs assessment, and an electrocardiogram (ECG).

Inclusion criteria were: (a) age 18–40 years, (b) absence of any major medical, endocrine, or neurological conditions, (c) no use of psychotropic medication, (d) good physical health, (e) a body mass index (BMI) between 18.5 and 28 kg/m², and (f) experience with psychostimulants in the past 12 months. Exclusion criteria included: (a) addiction, (b) history of psychiatric or neurological disorders, (c) pregnancy, (d) cardiovascular, gastrointestinal, hepatic, or renal abnormalities, (e) excessive smoking (> 15 cigarettes per day) or drinking (> 20 standard units of alcohol per week), (f) hypertension (diastolic > 90 mmHg; systolic > 140 mmHg), (g) serious side effects from previous psychostimulant use, and (h) being an active blood donor.

### Design and treatments

Data in the paper were recorded as part of a first- in -human, single-blind, placebo-controlled, cross-over trial. A full report on the safety data, including cardiovascular, psychostimulant, and psychotomimetic effects has been reported elsewhere (Ramaekers et al. 2024). Participants received single doses of 3-MMC (25, 50, and 100 mg) and placebo on separate days. 3-MMC was dissolved in approximately 150 mL of bitter lemon and administered orally, while the placebo consisted solely of bitter lemon. An escalating dosing scheme was employed, with participants receiving one of the following treatment sequences: 0–25–50–100 mg, 25–0–50–100 mg, 25–50–0–100 mg, or 25–50–100–0 mg. To avoid carry-over effects, each treatment was separated by a minimum washout period of 7 days. Participants were under medical supervision by a medical doctor during each 3-MMC administration day. The study was conducted in accordance with the ethical principles outlined in the Declaration of Helsinki (1964) and its subsequent amendments, and received approval from the Medical Ethics Committee of the Academic Hospital and Maastricht University. Additionally, a permit from the Dutch drug enforcement administration was obtained for the procurement, storage, and administration of 3-MMC. Participants were compensated monetarily for their involvement in the study. The trial was registered with the Dutch Central Committee on Research Involving Human Subjects (CCMO) under trial number NL84174.068.23 (NL-OMON53217).

### Procedures

Participants were instructed to abstain from drug use for at least one week before the study began and throughout its duration. Additionally, they were prohibited from consuming alcohol within 48 h, caffeine-containing beverages within 24 h, and tobacco before and during the experimental sessions. Participants were required to arrive well-rested. On test days, alcohol breath tests and urine drug screens were conducted upon arrival, to ensure compliance. The urine screens tested for the presence of opiates, cocaine, benzodiazepine, methamphetamine, amphetamine and tetrahydrocannabinol (THC). Treatments were administered only after confirming negative results from drug and alcohol screenings and, for female participants, after verifying a negative pregnancy test. Experimental procedures to induce and rate pain were repeated three times after dosing, i.e., at 45 min, 2 h and 30 min and 4 h and 30 min. A profile of mood questionnaire was administered at 1 and 5 h after dosing. Participants underwent a training session to familiarize themselves with the procedures and the experimental pain tests prior to study entrance.

### Pressure pain threshold (PPT)

The PPT task is a measure of deep muscular tissue sensitivity (Fischer 1987). The test determines the amount of pressure over a given area in which a steadily increasing non-painful pressure stimulus turns into a painful pressure sensation. An algometer (FDN 100, Wagner Instruments Inc., Greenwich, CT, USA) was used to deliver pressure on a skin area of 1 cm² between thumb and index finger of the left hand. The algometer has a force capacity (± accuracy) of 100 ± 2 N (10 ± 0.2 kgf) and graduation of 1 N (100 gf), respectively. A gradually increasing pressure was manually applied and participants were asked to indicate when the procedure became painful (pressure pain threshold, kg/cm²). Immediately after the end of the test, participants were asked to rate how painful, unpleasant and stressful the task was on 3 visual analogue scales (VAS) ranging from 0 (not at all) to 10 (extremely).

### Cold pressor test (CPT)

The CPT was used to induce a painful sensation according to previously validated procedures (Smeets et al. 2012). A tank was filled with water that was cooled to 3 °C. Participants were instructed to immerse their right hand in the cold water tank for as long as possible and to report the first occurrence of feeling pain. The maximal immersion duration was set to 3 min. Participants were not aware of this time limit. If the 3-minute maximum was achieved, the experimenter would signal the participant to remove the hand from the water. Dependent measures of the CPT included pain threshold (time to first report of pain) and pain tolerance (the number of seconds until withdrawal of the hand from the water tank). Immediately after the end of the test, participants were asked to rate painfulness, unpleasantness and stressfulness of the task on 3 visual analogue scales (VAS) ranging from 0 (not at all) to 10 (extremely).

### Profile of mood States (POMS)

The Profile of Mood States (POMS) is a self-assessment mood questionnaire with 72 items, rated on a 5-point Likert scale, from 0 (not at all) to 4 (extremely). Participants had to indicate to what extent these items were representative of their mood at that moment in time. Eight mood states were classified and quantified by calculating the sum score of associated items for each mood state, i.e. anxiety, depression, anger, vigor, fatigue, confusion, friendliness and elation. Two composite scales were derived, i.e., arousal ((anxiety-vigor)-(fatigue + confusion) and positive mood (elation– depression) (de Wit et al. 2002).

### Drug concentrations

Blood samples to quantify 3-MMC concentrations were taken repeatedly up to 5 h 30 min after dosing (Ramaekers et al. 2024). Here, we only present 3-MMC concentrations assessed at peak (1 h) and the end of the test session (5 h and 30 min). Blood samples were centrifuged, and serum was subsequently frozen at -30 °C. 3-MMC serum concentrations were quantified by a forensic routine analysis method (liquid-liquid extraction of 0.2 ml serum, analysis using liquid chromatography-tandem mass spectrometry (LC-MS/MS), 1 ng/ml lower limit of quantification).

### Statistics

The effects of 3-MMC on measures of pain perception and mood were analyzed by means of a Linear Mixed Model (LMM) with a restricted maximum likelihood method (REML). Model parameters included Dose, Time (after dosing) and Dose × Time as fixed effects, and a random intercept. Main effects of Dose were further evaluated by pairwise comparison between each 3-MMC dose and placebo (across time points). A compound symmetry covariance structure was used. The alpha criterion significance level was set at *p* =.05. All statistical tests were conducted with IBM SPSS version 27.0.

## Results

**Fig. 1:**
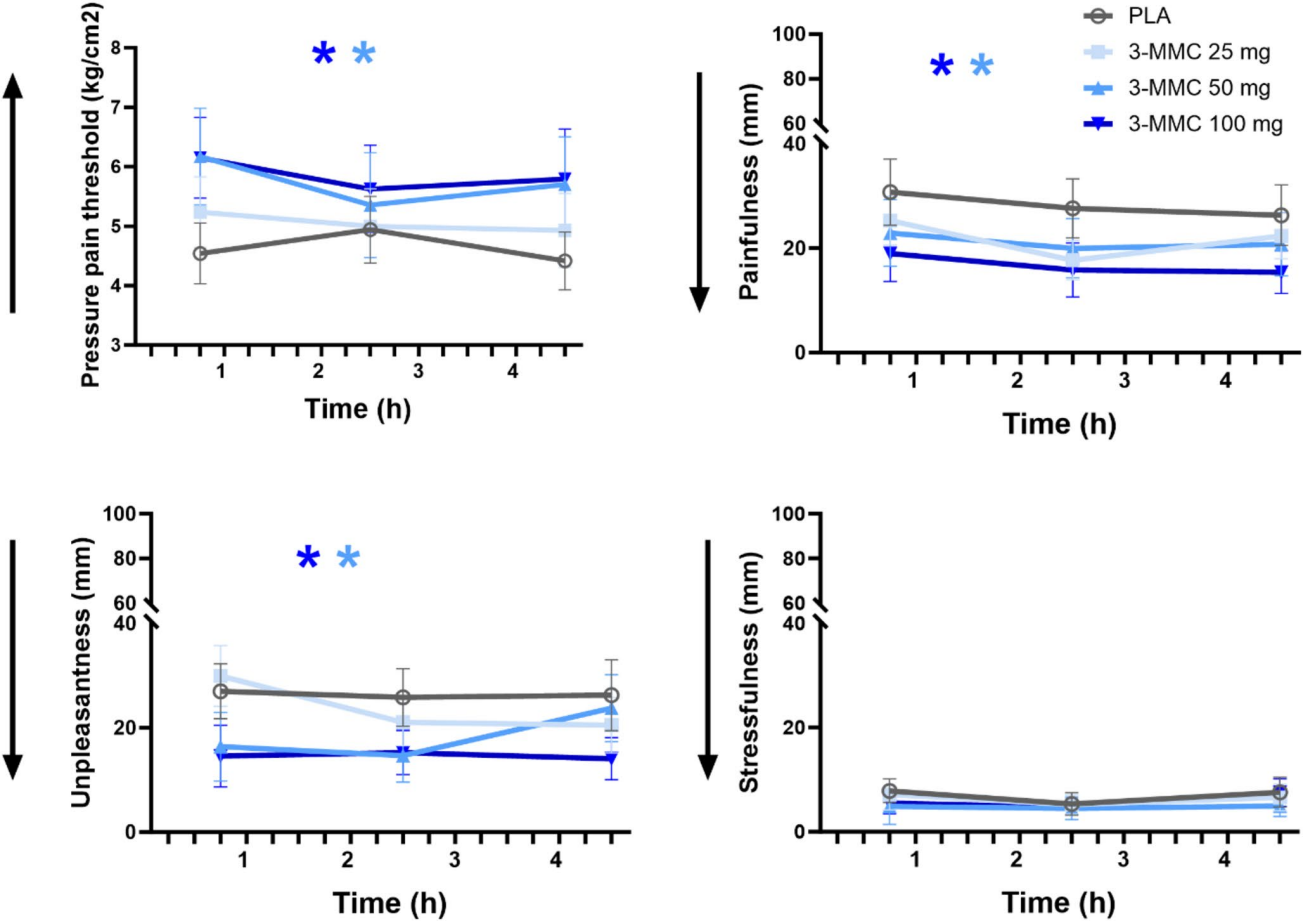
Mean (SE) pressure pain threshold and subjective ratings of painfulness, unpleasantness and stressfulness during the pressure pain threshold task (PPT) as a function of treatment condition and time after dosing. A * denotes a significant (*p* <.05) contrast between 3-MMC and placebo across different time points, with the color of the * representing the 3-MMC dose level. Arrows along the Y-axis indicate the direction of change that represents an analgesic effect

Figure 1 presents the mean (SE) objective and subjective ratings of pain perception during the PPT task in every treatment condition. LMM analyses showed main effects of Dose on pain threshold (F_3,128.450_=7.241, *p* <.001), painfulness (F_3,129.187_=5.586, *p* <.001) and unpleasantness (F_3, 129.399_=6.496, *p* <.001), but not on stressfulness. There was no main effect of Dose x Time for any of the main parameters in the PPT. Drug-placebo contrasts showed that 50 and 100 mg 3-MMC increased the pressure pain threshold (*p* =.011; *p* <.001) and reduced painfulness (*p* =.047; *p* <.001) and unpleasantness (*p* =.010; *p* <.001) during the PPT procedure.

**Fig. 2:**
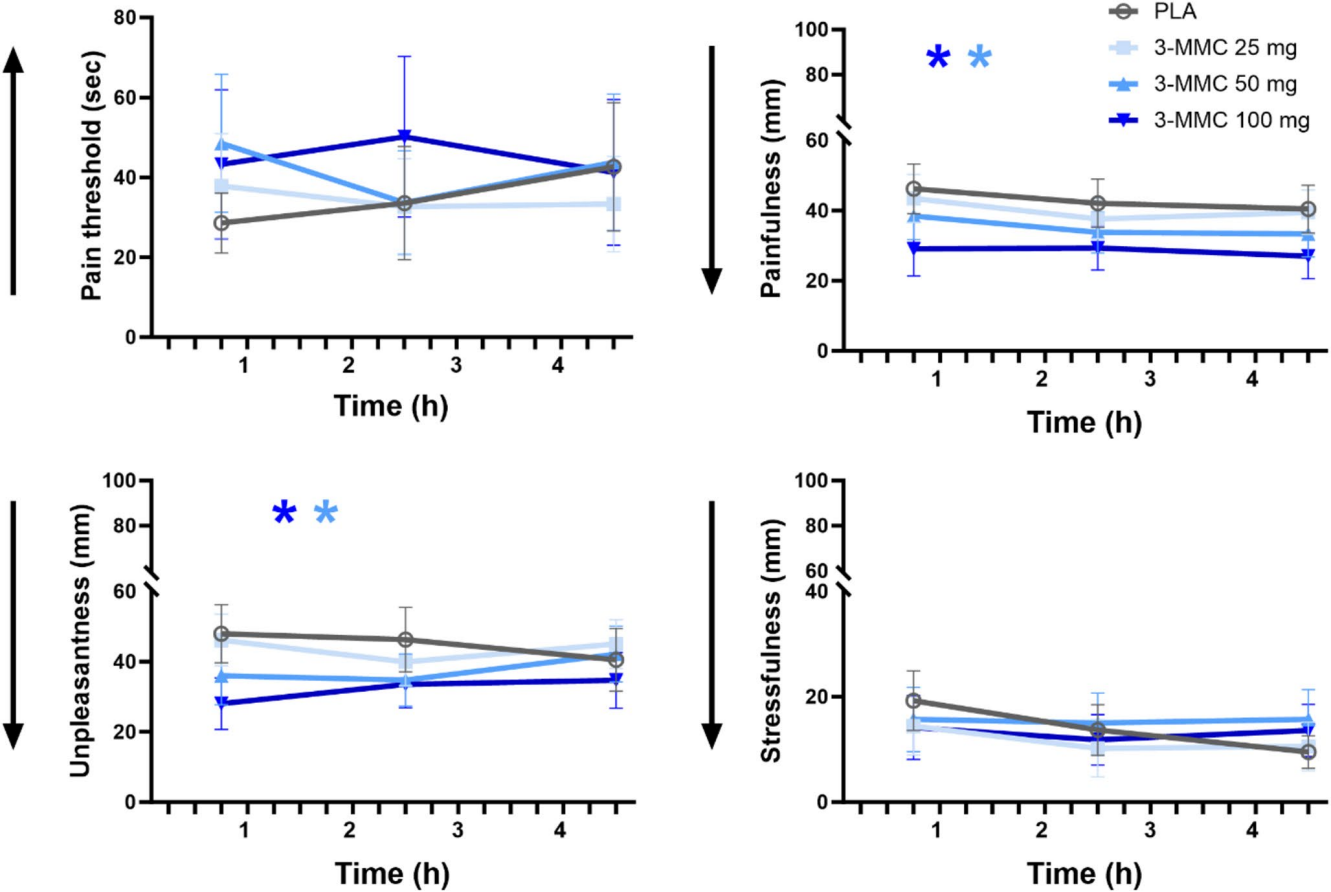
Mean (SE) pain threshold and subjective ratings of painfulness, unpleasantness and stressfulness during the cold pressor task (CPT) as a function of treatment condition and time after dosing. A * denotes a significant (*p* <.05) contrast between 3-MMC and placebo across different time points, with the color of the * representing the 3-MMC dose level. Arrows along the Y-axis indicate the direction of change that represents an analgesic effect

Figure 2 presents the mean (SE) objective and subjective ratings of pain perception during the CPT task in every treatment condition. LMM analyses showed main effects of Dose on painfulness (F_3, 128.887_=8.926, *p* <.001) and unpleasantness (F_3,128.722_=7.072, *p* <.001), but not on stressfulness and objective measures of pain threshold and pain tolerance. There was no Dose x Time interaction for any of the CPT measures. Drug-placebo contrasts revealed that 50 and 100 mg 3-MMC significantly reduced painfulness (*p* =.003; *p* <.001) and unpleasantness (*p* =.007; *p* <.001) during the CPT.

**Fig. 3:**
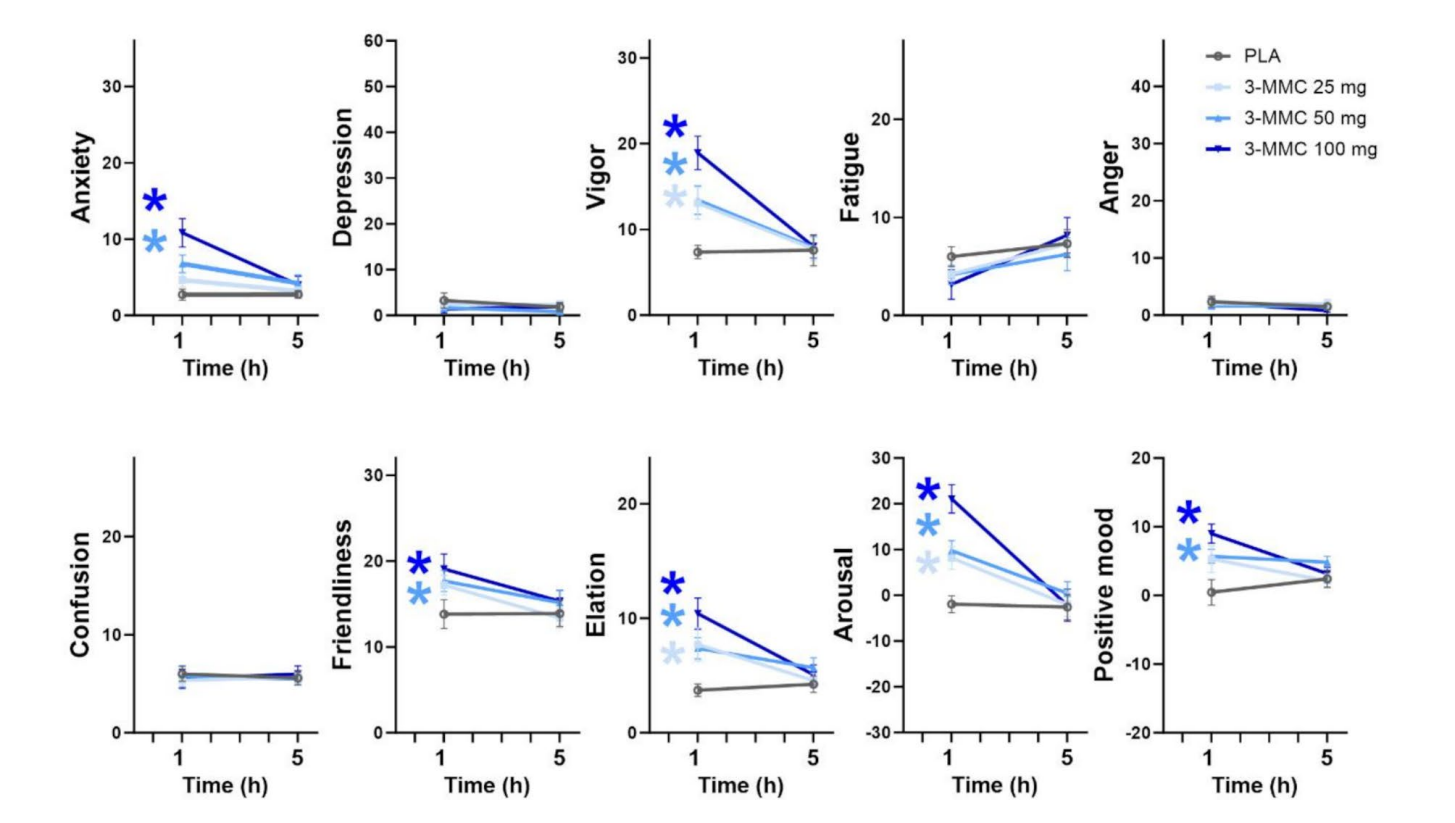
Mean (SE) ratings of mood as assessed with the Profile of Mood State (POMS) questionnaire as a function of treatment condition and time after dosing. A * denotes a significant (*p* <.05) contrast between 3-MMC and placebo across different time points, with the color of the * representing the 3-MMC dose level

Mean (SE) ratings of mood are shown in Fig. 3 for every treatment condition. LMM analyses showed significant main effects of Dose and Dose x Time for ratings of anxiety (F_3,82.134_=10.930, *p* <.001; F_3,80.770_=5.747, *p* <.001 ), vigor (F_3,81.503_=8.356, *p* <.001; F_3,80.350_=7.229, *p* <.001), elation (F_3,81.914_=7.935, *p* <.001; F_3,80.616_=4.847, *p* <.004), arousal (F_3,82.228_=9.500, *p* <.001; F_3,80.804_=8.703, *p* <.001) and positive mood (F_3,82.793_=5.497, *p* <.002; F_3,81.109_=3.602, *p* =.017). There was also a main effect of Dose on friendliness (F_3,81.538_=4.357, *p* <.007). Drug-placebo contrasts showed that 25 mg 3-MMC increased vigor (*p* =.018), elation (*p* =.004) and arousal (*p* =.017), while 50 and 100 mg 3-MMC increased anxiety (*p* =.002; *p* <.001), vigor (*p* =.011; *p* <.001), friendliness (*p* =.007; *p* =.001), elation (*p* <.001; *p* <.001), arousal (*p* =.001; *p* <.001) and positive mood (*p* =.002; *p* <.001).

Mean, dose-related (SD) 3-MMC peak concentrations at 1 h after dosing were 29(12), 75 (40) and 198 (71) ng/mL after 25, 50 and 100 mg respectively. At 5.5 h after dosing, mean (SD) 3-MMC concentrations were 18 (9), 41 (20) and 76 (34) ng/mL after the 25, 50 and 100 mg dose, respectively. Peak concentrations (C_max_) were reached in median at 1.5 h after ingestion (range 0.75–3.0 h) and the estimate for elimination was a median half-life of 3.0 h (range 1.5–6.0 h, interquartile range 2.5–3.6 h) (Ramaekers et al. 2024).

## Discussion

The present study aimed to assess the analgesic effects of low to moderate doses (25, 50 and 100 mg) of 3-MMC in healthy volunteers who were exposed to experimentally induced pain. Overall, 3-MMC produced dose-related elevations in the pressure pain threshold and reduced subjective experience of pain, in both experimental pain models, that persisted consistently for up to 5 h after dosing. 3-MMC also produced dose-related increments in mood that were prominent at 1 h after dosing but not at 5 h after dosing.

3-MMC induced dose-related analgesic effects as assessed with objective and subjective measures of pain across two experimental pain paradigms. Subjectively, participants rated levels of painfulness and unpleasantness of pain induced in the PPT and CPT to be lower after a single dose of 50 and 100 mg. Objectively, 3-MMC increased the pain threshold in the PPT, particularly after the 50 and 100 mg dose. In the CPT, mean pain threshold also increased after the 100 mg dose but that change did not reach statistical significance, possibly because variance in task performance in the CPT is intrinsically high. There were no Dose x Time interaction effects in any of the experimental pain paradigms, indicating that the analgesic effect of 3-MMC persisted up to 5 h after dosing, despite falling 3-MMC concentrations. It is likely therefore that the analgesic effects of 3-MMC also sustained beyond the 5-hour observation window of the present study. Indeed, preclinical work has shown that cathinones can produce analgesic effects in a tail-flick test that last for 24 h (Nencini et al. 1984). In the present study, 3-MMC concentrations dropped considerably between peak concentrations at about 1 h and a subsequent measurement at 5.5 h consistent with an estimated median elimination half-life of 3.1 h (range 1.5–6 h). This suggests that total clearance of 3-MMC could take up to a day. This would be in line with the notion from preclinical work that analgesic effects of single doses of a cathinone may pertain for a prolonged period of time.

On average, the magnitude of the analgesic effect of 3-MMC at 50 and 100 mg as shown by a reduction of subjective pain perception and an increase in pressure pain threshold varied between 20 and 40%, as compared to placebo. These effects appear similar to or even larger than some of the analgesic effects that have been observed with opioids in healthy volunteer studies using the same experimental pain manipulations. For example, an oral dose of morphine (30 mg) increased the pain threshold as assessed with the PPT and the CPT by around 10% (Naef et al. 2003), while intravenous infusions of buprenorphine (0.6 mg) and morphine (20 mg) increased pressure pain threshold by about 20–25% (Ravn et al. 2013). Likewise, oral doses of codeine (60 and 120 mg), morphine (20 or 40 mg) and oxycodone (20 mg) reduced subjective pain experience by about 10–30% during the CPT (Cooper et al. 2012; Walker and Zacny 1998). Amphetamine like compounds such as methylphenidate (20 mg) increased pain tolerance in the CPT by up to 50% in healthy volunteers (Pud et al. 2017) and in patients suffering from Attention Deficit Hyperactivity Disorder (Treister et al. 2015). These observational comparisons of analgesic effects of opioids, methylphenidate and 3-MMC suggest that the magnitude of analgesic effects produced by 3-MMC potentially are of clinical relevance.

The analgesic effect of 3-MMC occurred independent from its subjective effects on mood. 3-MMC increased subjective ratings of arousal and vigor as well as ratings of positive mood, most notably elation and friendliness, in a dose-related manner. Increments in mood however were prominent at 1 h after dosing but absent at 5 h after dosing as indicated by a significant Dose x Time interaction. The finding that subjective effects of 3-MMC dissipated within 5 h after dosing, while the analgesic effect of 3-MMC pertained, suggests differential mechanisms of action underlying both phenomena. Preclinical work has shown that cathinone-induced analgesia primarily depends on the stimulation of monoaminergic pathways, followed by a release of opioid peptides. Involvement of monoaminergic pathways was concluded from the finding that the analgesic effect of cathinone could be prevented by reserpine and p-chlorophenylalanine, which deplete catecholamines (noradrenaline and dopamine) and serotonin respectively (Nencini et al. 1984). Involvement of the opioid system was deduced from the findings that cathinone increases morphine-induced analgesia, and that this entire effect could be blocked by naltrexone, a potent opioid antagonist (Della Bella et al. 1985; Nencini et al. 1984). Stimulant effects of cathinones on arousal and mood on the other hand are generally believed to result from acute increments in dopamine, noradrenaline and serotonin (Kelly 2011), irrespective of cathinone-induced changes in opioid signaling. It can be speculated therefore that temporal differences in analgesic and subjective effects of 3-MMC result from the involvement of the opioid system in the former, but not in the latter. Yet, effects of cathinones on monoamine and opioid transmission might still be interconnected as cathinones do not interact directly with opioid receptors (Luethi et al. 2018).

While the current finding of 3-MMC’s analgesic properties supports earlier insights into the inherent analgesic qualities of amphetamines and related compounds (Bustamante et al. 2004; Dalal and Melzack 1998; Nencini et al. 1984), its translation to clinical applications remains a distant prospect for a number of reasons. First, it is not entirely clear which clinical pain conditions are modeled with the PPT and the CPT. Pressure algometry is the most commonly used technique for quantifying pain. It activates group III and IV afferent nerves, and might serve as an experimental counterpart to pain upon palpation (e.g., abdomen or back) in clinical practice (Staahl et al. 2009). The cold pressor test is known to activate the “diffuse noxious inhibitory control system”, a network of descending neuronal pathways originating in the brainstem that provides negative feedback to modulate incoming pain signals at the spinal cord level, and which is impaired in many functional pain syndromes (Staahl et al. 2009). It is important to recognize however that experimental pain activates only a portion of the complex mechanisms involved in pathological pain, which limits the ability to translate analgesic effects observed in experimental settings into clinical outcomes. The clinical utility of the use of 3-MMC and other cathinones therefore should be further evaluated in clinical trials in patients with specific pain syndromes. Second, analgesic effects assessed with the CPT might be sensitive to expectancy effects (Devlin et al. 2019). Expectancies may have built up in the present study in which all subjects were exposed to 3-MMC at incremental dose steps. The present findings should therefore be replicated in placebo controlled trials in which treatment orders are balanced. Third, the potential of stimulants like 3-MMC in the treatment of pain disorders might be outbalanced by the risks of adverse events, abuse and dependence. It should be noted however that abuse potential and adverse events have been primarily associated with the use of high doses of 3-MMC, often in combination with other drugs (Backberg et al. 2015; EMCDDA 2023). Low to moderate doses of 3-MMC for which we evaluated the analgesic effects in this study, did not increase ratings of drug liking and wanting at 5 h after dosing (Ramaekers et al. 2024). Likewise, cardiovascular, psychostimulant and psychotomimetic effects of 3-MMC at low to moderate doses were generally mild and well tolerated (Ramaekers et al. 2024). This seems to suggest that the adverse event profile of 3-MMC is benign at low to moderate doses which might increase opportunities for medical use of 3-MMC in the treatment of pain. Given its mood enhancing property, 3-MMC might also provide a potential treatment for mental health disorders related to pain such as premenstrual dysphoric disorder (PMDD). In order to reduce the risk of abuse and dependence, it should be preferred to assess the analgesic properties of 3-MMC in acute or transient pain conditions, e.g. rather than in the treatment of chronic pain.

In summary, 3-MMC produced analgesic effects at doses that appear low enough to avoid challenging subjective experiences and that have been associated with a benign side effect profile. The present data warrant further research into the analgesic effects of low to moderate doses of 3-MMC in patient populations.

## Data Availability

Na.
